# Supplementary figures and images for: Weirdo19ES is a novel singleton mycobacteriophage that selects for glycolipid deficient phage-resistant M. smegmatis mutants
Source: PLoS One. 2020 May 1;15(5):e0231881. doi: 10.1371/journal.pone.0231881 (PMC7194413; doi:10.1371/journal.pone.0231881)

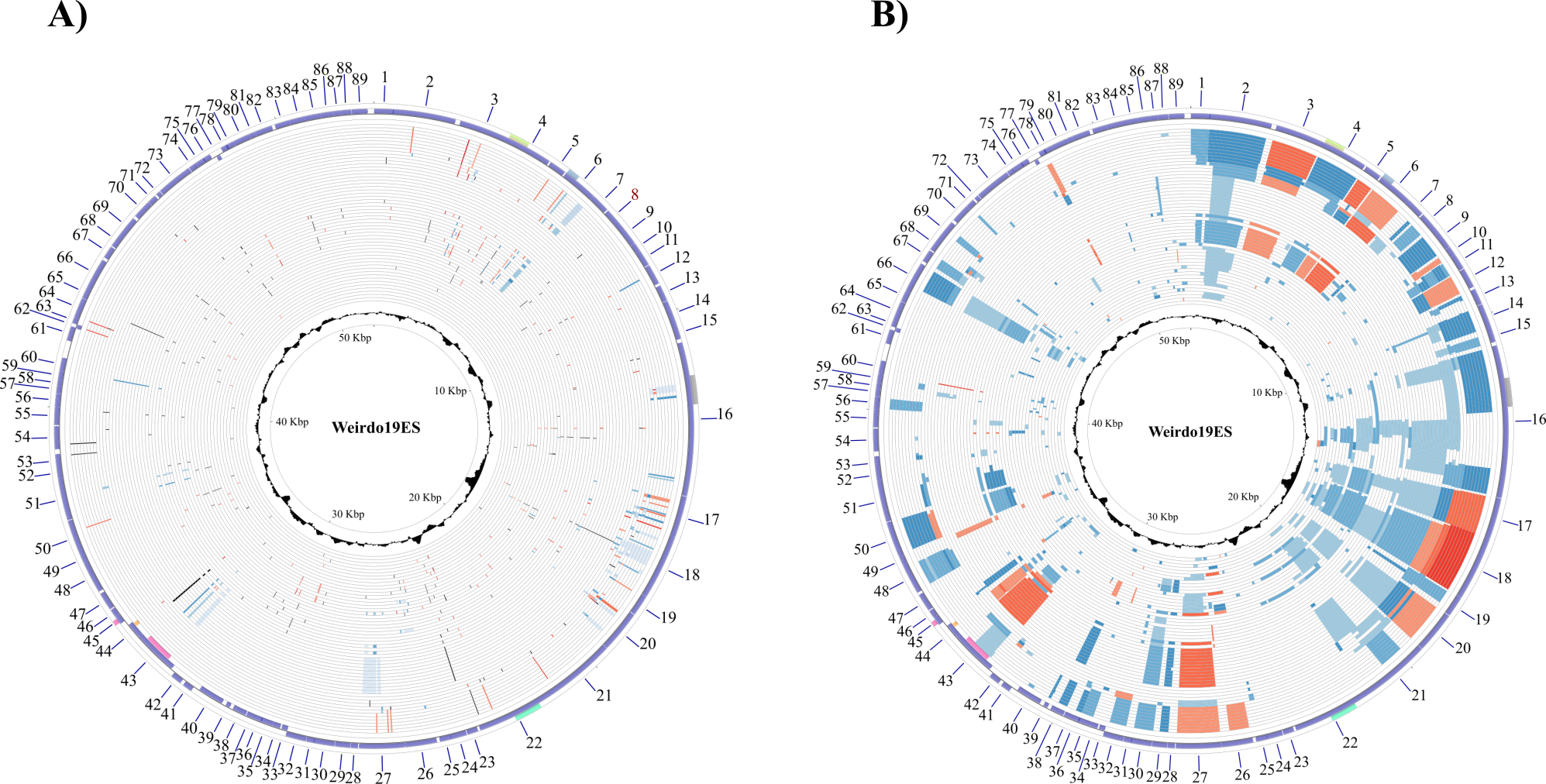

Supplement: S1 Fig — Circular maps comparing Weirdo19ES with a set of bacteriophages of different clusters infecting either Gordonia or Mycobacterium hosts are displayed. The outermost ring corresponds to the reference genome and the internal rings shows the regions of BLASTN and BLASTP homology, A and B, respectively. The inner ring displays the GC content of Weirdo19ES. (TIF) [file pone.0231881.s001.tif]

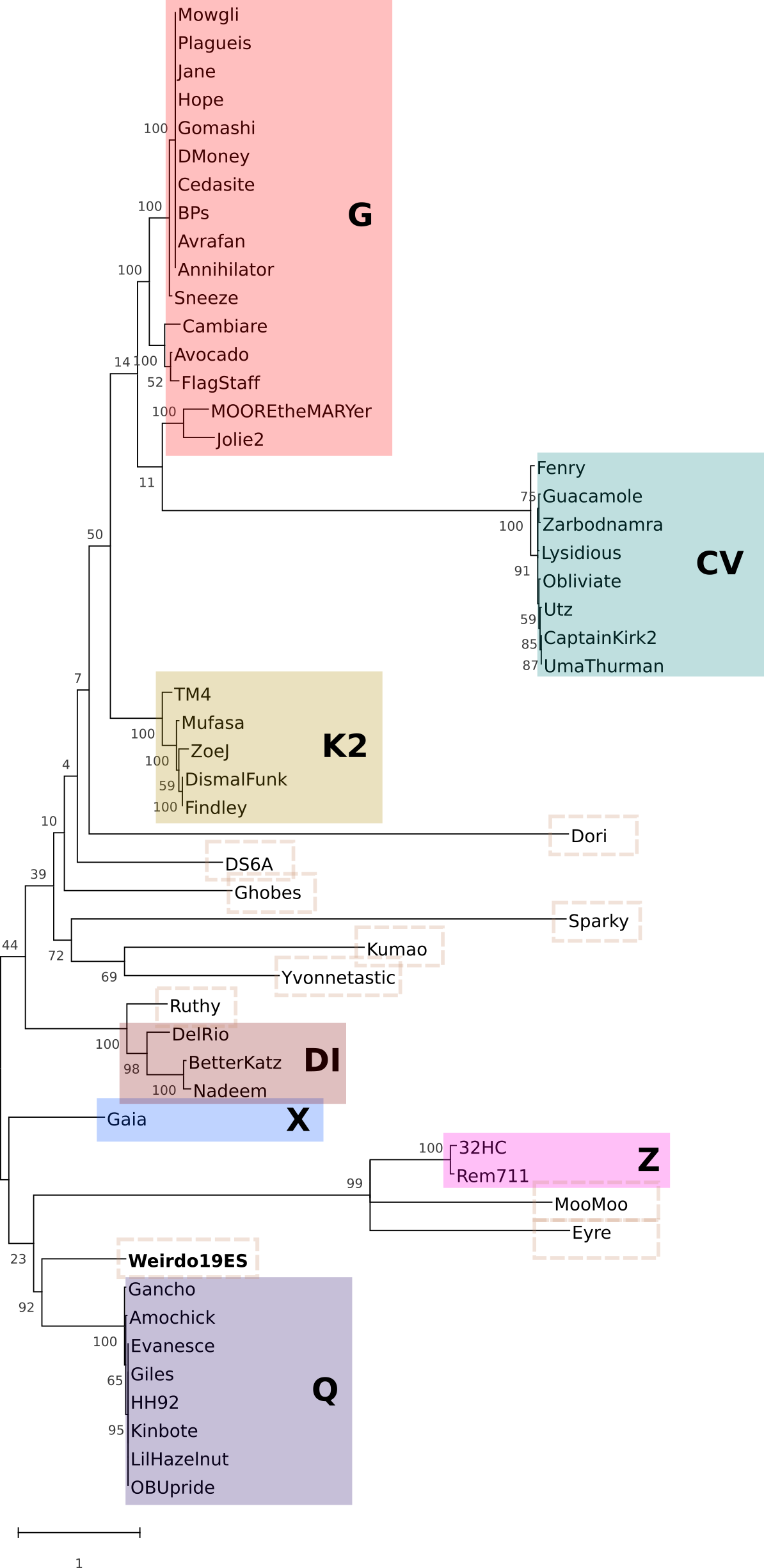

Supplement: S2 Fig — The amino acid sequences of the TMPs were aligned with MUSCLE and the dendrogram was constructed using Maximum Likelihood method in MEGAX: mycobacteriophage clusters are indicated in colored boxes. (TIF) [file pone.0231881.s002.tif]
