# Supplementary material for: Weirdo19ES is a novel singleton mycobacteriophage that selects for glycolipid deficient phage-resistant M. smegmatis mutants
Source: PLoS One. 2020 May 1;15(5):e0231881. doi: 10.1371/journal.pone.0231881 (PMC7194413; doi:10.1371/journal.pone.0231881)
Supplement: S1 Table — Corresponding accession numbers, host and clusters classification are listed. (DOCX) [file pone.0231881.s003.docx]

| **Bacteriophage** | **Host** | **Cluster** | **Accession number** |
| --- | --- | --- | --- |
| 32HC | *M. smegmatis mc^2^155* | Z | KJ028219 |
| AMOCHICK | *M. smegmatis mc^2^155* | Q | MH697577 |
| ANNIHILATOR | *M. smegmatis mc^2^155* | G1 | KT365399 |
| AROOSTOOK | *M. smegmatis mc^2^155* | G1 | MF668268 |
| AVOCADO | *M. smegmatis mc^2^155* | G2 | MF141540 |
| AVRAFAN | *M. smegmatis mc^2^155* | G1 | JN699002 |
| BETTERKATZ | *Gordonia terrae 3612* | DI | KU963261 |
| BPS | *M. smegmatis mc^2^155* | G1 | EU568876 |
| CAMBIARE | *M. smegmatis mc^2^155* | G2 | KR080198 |
| CAPTAINKIRK2 | *Gordonia terrae 3612* | CV | KX557274 |
| CEDASITE | *M. smegmatis mc^2^155* | G1 | KT355472 |
| DELRIO | *Gordonia terrae 3612* | DI | MH509446 |
| DISMALFUNK | *M. smegmatis mc^2^155* | K2 | MF140408 |
| DMONEY | *M. smegmatis mc^2^155* | G1 | MH371116 |
| DORI | *M. smegmatis mc^2^155* | SINGLETON | JN698995 |
| DS6A | *M. tuberculosis H37Rv* | SINGLETON | JN698994 |
| EVANESCE | *M. smegmatis mc^2^155* | Q | KT454972 |
| EYRE | *Gordonia terrae 3612* | SINGLETON | KX557277 |
| FENRY | *Gordonia terrae 3612* | CV | MH020241 |
| FINDLEY | *M. smegmatis mc^2^155* | K2 | MF140411 |
| FLAGSTAFF | *M. smegmatis mc^2^155* | G2 | KR080197 |
| FROSTY24 | *M. smegmatis mc^2^155* | G1 | KT355474 |
| GAIA | *M. smegmatis mc^2^155* | X | KJ567043 |
| GANCHO | *M. smegmatis mc^2^155* | Q | MH727549 |
| GHOBES | *Gordonia terrae 3612* | SINGLETON | KX557278 |
| GILES | *M. smegmatis mc^2^155* | Q | EU203571 |
| GOMASHI | *M. smegmatis mc^2^155* | G1 | KM923970 |
| GUACAMOLE | *Gordonia terrae 3612* | CV | KU963259 |
| HH92 | *M. smegmatis mc^2^155* | Q | KJ538722 |
| HOPE | *M. smegmatis mc^2^155* | G1 | GQ303261 |
| JANE | *M. smegmatis mc^2^155* | G1 | KX588251 |
| JOLIE2 | *M. smegmatis mc^2^155* | G4 | KJ410133 |
| KINBOTE | *M. smegmatis mc^2^155* | Q | KT222940 |
| KUMAO | *M. smegmatis mc^2^155* | SINGLETON | MG009575 |
| LILHAZELNUT | *M. smegmatis mc^2^155* | Q | MF919517 |
| LYSIDIOUS | *Gordonia terrae 3612* | CV | MF919521 |
| MOOMOO | *M. smegmatis mc^2^155* | SINGLETON | MH001449 |
| MOORETHEMARYER | *M. smegmatis mc^2^155* | G3 | KR080202 |
| MOWGLI | *M. smegmatis mc^2^155* | G1 | MH479920 |
| MUFASA | *M. smegmatis mc^2^155* | K2 | KT591490 |
| NADEEM | *Gordonia terrae 3612* | DI | MH399781 |
| OBLIVIATE | *Gordonia terrae 3612* | CV | KU963254 |
| OBUPRIDE | *M. smegmatis mc^2^155* | Q | KT246485 |
| PLAGUEIS | *M. smegmatis mc^2^155* | G1 | MH450127 |
| REM711 | *M. smegmatis mc^2^155* | Z | MG770216 |
| RUTHY | *Gordonia terrae 3612* | SINGLETON | MH536826 |
| SNEEZE | *M. smegmatis mc^2^155* | G1 | KX534004 |
| SPARKY | *M. smegmatis mc^2^155* | SINGLETON | KM083128 |
| TM4 | *M. smegmatis mc^2^155* | K2 | AF068845 |
| UMATHURMAN | *Gordonia terrae 3612* | CV | KU963251 |
| UTZ | *Gordonia terrae 3612* | CV | KU998248 |
| YVONNETASTIC | *Gordonia terrae 3612* | SINGLETON | KU963248 |
| ZARBODNAMRA | *Gordonia terrae 3612* | CV | MH576969 |
| ZOEJ | *M. smegmatis mc^2^155* | K2 | KJ510412 |
