# Supplementary material for: Weirdo19ES is a novel singleton mycobacteriophage that selects for glycolipid deficient phage-resistant M. smegmatis mutants
Source: PLoS One. 2020 May 1;15(5):e0231881. doi: 10.1371/journal.pone.0231881 (PMC7194413; doi:10.1371/journal.pone.0231881)
Supplement: S2 Table — The function were predicted by BLASTP analysis using phagedb database. (1) The highest homologue to each gene product are listed together with the phage cluster and BLASTP E-value (2). (DOCX) [file pone.0231881.s004.docx]

| **Gene** | **Predicted function** | **BLASTP homolog^(1)^** | **Cluster** | **E-value^(2)^** |
| --- | --- | --- | --- | --- |
| **1** | Terminase small subunit | Gancho_1 | Q | 1e-23 |
| **2** | Terminase large subunit | Rem711_3 | Z | 1e-106 |
| **3** | Portal protein | Gancho_5 | Q | 1e-144 |
| **4** | Capsid maturation protease | Webster2_Draft_7 | Q | 1e-74 |
| **5** | Scaffolding protein | Webster2_Draft_8 | Q | 2e-43 |
| **6** | Major capsid protein | WheatThin_Draft_10 | DI | e-112 |
| **7** | Head-tail connector | Webster2_Draft_10 | Q | 2e-07 |
| **8** | Head-tail connector | Webster2_Draft_11 | Q | 2e-11 |
| **9** | Head-tail connector | Rem711_15 | Z | 1e-41 |
| **10** | Head-to-tail connector protein | Rem711_16 | Z | 2e-12 |
| **11** | Head-to-tail connector complex protein | Gancho_13 | Q | 1e-20 |
| **12** | Major tail subunit | Webster2_Draft_15 | Q | 3e-58 |
| **13** | Function unkwon | Low homology |  |  |
| **14** | Tail assembly chaperone | 32HC_22 | Z | 2e-46 |
| **15** | Tail assembly chaperone | Rem711_22 | Z | 3e-10 |
| **16** | Tape measure protein | Nebkiss_13 | X | 1e-144 |
| **17** | Minor tail subunit | Webster2_Draft_21 | Q | 1e-147 |
| **18** | Minor tail protein | Gancho_21 | Q | 0.0 |
| **19** | Minor tail protein | Webster2_Draft_23 | Unclustered | 1e-18 |
| **20** | Minor tail protein | Daegal_Draft_25 | Q | 1e-124 |
| **21** | Minor tail protein | Adjutor_31 | D1 | 1e-123 |
| **22** | D-Ala-D-Ala carboxypeptidase  / minor tail protein | Nova_31 | D1 | 1e-113 |
| **23** | Function unkwon | Helpful_Draft_34 | D1 | 7e-30 |
| **24** | Function unkwon | WaldoWhy_Draft_35 | D1 | 5e-47 |
| **25** | Function unkwon | Low homology |  |  |
| **26** | Lysin A | DS6A_30 | Singleton | 4e-99 |
| **27** | Lysin B | Gideon_28 | G1 | 1e-161 |
| **28** | Holin | Nebkiss_36 | X | 1e-11 |
| **29** | Function unknown | Serendipitous_17 | B5 | 7e-20 |
| **30** | Function unknown | Cooper_17 | B4 | 7e-33 |
| **31** | Function Unknown | FlagStaff_55 | G2 | 2e-05 |
| **32** | Virion protein | Rem711_41 | Z | 7e-35 |
| **33** | Function Unknown | Low homology |  |  |
| **34** | Function Unknown | Rem711_85 | Z | 5e-05 |
| **35** | Function Unknown | Webster2_Draft_41 | Q | 6e-11 |
| **36** | Function Unknown | Webster2_Draft_42 | Q | 9e-09 |
| **37** | Function Unknown | Low homology |  |  |
| **38** | Function unknown | Milly_39 | K2 | 9e-27 |
| **39** | Function Unknown | Low homology |  |  |
| **40** | Function unknown | StevieRay_54 | P1 | 4e-64 |
| **41** | Function Unknown | Low homology |  |  |
| **42** | Function Unknown | 32HC_8 | Z | 2e-12 |
| **43** | Integrase | Grizzly_32 | G1 | 1e-124 |
| **44** | Immunity repressor | Sneeze_33 | G1 | 2e-35 |
| **45** | Excisionase | Lemuria_Draft_33 | G4 | 2e-13 |
| **46** | Function Unknown | Low homology |  |  |
| **47** | Function Unknown | Low homology |  |  |
| **48** | HTH DNA-binding protein | 32HC_51 | Z | 4e-34 |
| **49** | Function Unknown | Paito_41 | G1 | 8e-16 |
| **50** | Exonuclease-like protein | Rem711_53 | Z | 2e-77 |
| **51** | ssDNA binding protein | William_Draft_61 |  | 6e-55 |
| **52** | Function Unknown | Low homology |  |  |
| **53** | Function unknown | Cracklewink_Draft_66 | Y | 2e-04 |
| **54** | Function unknown | Fionnbharth_55 | K4 | 1e-07 |
| **55** | Function unknown | MarkPhew_Draft_2 | K1 | 6e-05 |
| **56** | Function unknown | Webster2_Draft_62 | Q | 9e-09 |
| **57** | No hits found | Low homology |  |  |
| **58** | Function unknown | Indlovu_Draft_93 | B(B4) | 8e-05 |
| **59** | Function unknown | 32HC_65 | Z | 2e-06 |
| **60** | Function unknown | Wamburgrxpress_60 | L1 | 2e-23 |
| **61** | HNH endonuclease | Comrade_60 | BK1 (*Streptomyces* phage) | 5e-13 |
| **62** | No hits found |  |  |  |
| **63** | Function unknown | Quico_85 | F1 | 9e-04 |
| **64** | Function Unknown | Low homology |  |  |
| **65** | Function Unknown | Low homology |  |  |
| **66** | RuvC-like resolvase | Giles_67 | Q | 2e-38 |
| **67** | WhiB-like transcriptional regulator | Webster2_Draft_75 | Q | 5e-10 |
| **68** | Function Unknown | Apizium_62 | B1 | 4e-14 |
| **69** | function unknown | Rem711_74 | Z | 6e-05 |
| **70** | Function Unknown | Low homology |  |  |
| **71** | Function Unknown | Low homology |  |  |
| **72** | No hits found | Low homology |  |  |
| **73** | Function unknown | Terror_48 | G1 | 2e-12 |
| **74** | function unknown | Bipper_132 | Y | 1e-10 |
| **75** | Function Unknown | Low homology |  |  |
| **76** | Function Unknown | Low homology |  |  |
| **77** | No hits found |  |  |  |
| **78** | Function Unknown | WheatThin_Draft_73 | DI | 3e-08 |
| **79** | Function Unknown | Low homology |  |  |
| **80** | Function Unknown | Low homology |  |  |
| **81** | Function unknown | Low homology |  |  |
| **82** | Function Unknown | Turuncu_26 | CR (*Gordonia* phage) | 7e-06 |
| **83** | Function Unknown | Low homology |  |  |
| **84** | Function unknown | BigNuz_82 | P1 | 1e-19 |
| **85** | Function Unknown | Low homology |  |  |
| **86** | Function unknown | ThulaThula_Draft_68 | P | 9e-06 |
| **87** | Function Unknown | Low homology |  |  |
| **88** | Function Unknown | Shyg_38 | BC1 (*Streptomyces* phage) | 2e-05 |
| **89** | HNH endonuclease | KaiHaiDragon_54 | EC (*M. foliorum*) | 3e-16 |
