# Supplementary material for: Weirdo19ES is a novel singleton mycobacteriophage that selects for glycolipid deficient phage-resistant M. smegmatis mutants
Source: PLoS One. 2020 May 1;15(5):e0231881. doi: 10.1371/journal.pone.0231881 (PMC7194413; doi:10.1371/journal.pone.0231881)
Supplement: Attachment — Submitted filename: PONE-D-19-31407.docx [file pone.0231881.s005.docx]

**PONE-D-19-31407**

**Weirdo19ES is a novel singleton mycobacteriophage that selects for glycolipid deficient phage resistant Mycobacterium smegmatis mutants**

This is a nice attempt by Cristian Alejandro Suarez and coauthors to characterize singleton mycobacteriophage that selects for glycolipid deficient phage resistant *Mycobacterium smegmatis* mutants. The authors have compiled large set of bioinformatics data for mycobacteriophage. This characterized mycobacteriophage Weirdo19ES is adding new information and opening research possibilities on the role of mycobacterial GPLs as receptors. On through review of the manuscript, I found several errors which need to be corrected before acceptance for publication in the journal.

**Comments:**

- The english is very poor, and grammatically incorrect. It’s hard to understand the statements made by authors, as those are too long and complex. Please be consistent in using nomenclature (Use Weirdo19ES or 19WeirdoES, similarly Maximum Likelyhood method or Maximum Likelihood method). Through rewriting with grammar corrections is required for possible acceptance.
- Figures are with compromised resolutions, hardly readable and difficult to understand the message. Fonts and figures are not appropriate for publication.
- In line 226, 70,19% GC content need to be corrected with 70.19%. Similarly, in line 227, 96,1% should be 96.1%.
- Analysis and homology of the LysA needs to be revalidated. In Fig 2A, both Che9C and DS6A are similar in domain organization, as they belong to same group Org-X. But in Fig. 2B, homology has been studied only with DS6A and other genomes? Why not Che9C is included in this study? If it was included, what is the homology with Weirdo19ES? If it has homology, then why not to say that Weirdo19ES is similar to Org-X group. In Fig. 2B, it looks like SG4 and some other genomes also have homology with Weirdo19ES. Please include these other homology.
- In line 269, what is the significance of “Nonstructural genes ORFs 33-40”?
- In genome organization (line 234), ORF 33-40, 43, 44, 62 and 77 are mentioned to be transcribed leftward. There is no information about gene 45 in this part, but in integration and immunity region (line 283), gene 45 is included as a part of ORF 43-45. What is the orientation of gene 45, it is leftward or rightward?
- Statement in line 235 and 236 is that gene 43 (int) and gene 44 (rep) are transcribed leftward, while in line 314, 315 and 316, statement tells that int and rep are transcribed rightward. This kind of statements are due to shear negligence and must be taken care prior submission.
- In line 285, phages BPs, Halo, Annihilator and Avrafan are mentioned having relationship with Weirdo19ES, but in Fig.3A, Annihilator is missing.
- In Fig. 3A on the basis of integrase, homology is reported with BPs, Halo, Annihilator, Avrafan, UmaThurman, cluster CVs and Y. In Fig. 4, only BPs, UmaThurman and Bipper are included for intervening region and ssrA tag study. What was the basis to select these members only?
- Similarly, on the basis of repressor, homology/ similarity was with cluster Y, not with G1. Bipper is from cluster Y, but in Fig.4B, BPs also have that conserved alanine. What is the conclusion of this observation?
- In Fig. 4B, colour of highlight is not visible.
- Heading in line no 334 has error.
- In line 340, ORF 89 is predicted to be HNH using InterProScan. In line 345, ORF 61 is proposed to be HNH, using HMMER. It is not clear that both ORFs (89 and 61) are HNH? Why authors used two different platforms and predicted two different HNH.
- In comparative genomics study, why group Y (significantly similar in repressor based study) was not included and why K2, Q, D1, G2, G3 and G4 were included as these were not included or mentioned in any other study of the article.
- In line 411, authors stated that Weirdo19ES, Q and Z are more related to CV & D1. But what about intermediate values of G1, G4, G2, G3?
- According to authors G is distant, it is surprising as it has lower values than CV.
- In line 465, it is 102 CFU or 10^2^ CFU?
- Legend of Fig.11 says GLP, while Figure denotes GPL.
- Pictures are not clear in Fig.11 A and B. What was the loading control?
- Conclusion portion must be summarised.
