# Supplementary material for: Weirdo19ES is a novel singleton mycobacteriophage that selects for glycolipid deficient phage-resistant M. smegmatis mutants
Source: PLoS One. 2020 May 1;15(5):e0231881. doi: 10.1371/journal.pone.0231881 (PMC7194413; doi:10.1371/journal.pone.0231881)
Supplement: Attachment — Submitted filename: PONE-D-19-31407_Comments.docx [file pone.0231881.s006.docx]

In this manuscript titled “Weirdo19ES is a ……………………………*Mycobacterium smegmatis* mutants” Suarez and co-workers describe the genomic features of a singleton Mycobacteriophage Weirdo19ES by using various bioinformatics tools. They further described the possible receptor for this phage on *M. smegmatis* surface and demonstrated that cell surface polar GLPs act as receptor. I must admit that authors have done a very thorough analysis of this new mycobacteriophage and their study is well designed and executed. However, there are few issues with the manuscripts;

Major issues:

1. While the *M. smegmatis* GLP deficient mutants are resistant to Weirdo19ES the experiments performed here do not establish direct interaction of Phage to GLPs on bacterial cells. It will be advisable to do an experiment where Phage is adsorbed on purified GLPs before being used in the plaque assay. In the absence of sufficient quantity of purified GLPs same experiment can be performed using heat killed 1R-Weirdo19ES and wild type MC^2^155.
2. The manuscript needs careful proofreading for correcting grammatical and typographical mistakes. I have highlighted some of the examples in the minor issues section below. However, this is not the comprehensive list of language related mistakes. I strongly encourage the authors to have the manuscript proofread for language from their English proficient colleagues.

Minor issues:

Please remember this is a small list of typographical and grammatical mistakes and the entire manuscript needs to be carefully read and mistakes need to be corrected.

1. Throughout the manuscript punctuations after the reference are added after space, remove them.
2. At several places a hyphen (-) is added before a word for no reason, remove them.
3. At some places a comma (,) is used instead of the decimal (.)
4. Line 41: mycobacteriophage ~~Patience~~ *from* patient
5. Lines 43-44: ~~In the same scenario, analysis of phages infecting Gordonia species showed similarities to mycobacteriophages [4]~~ (not adding anything)
6. Line 47: It ~~called~~ caught our attention ~~the fact~~ that uncommon
7. Line 79: Preparation ~~and~~ of
8. Line 80: briefly late log ~~fresh~~ phase
9. Line 133: Is it true that the images for colony morphology were taken at 0.8X magnification? Or should it be 8X?
10. Line 152: Was 0.5% glycerol in the medium was taken by weight (w/v) or is it a typographical mistake?
11. Line 168: Provide reference for Jacobs and Hatfull protocol.
12. Line 212: ~~The~~ To this
13. Line 334: genome ~~if~~ of Weirdo19ES
14. Change the title of the last section from Conclusion to Discussion and remove Discussion from Results and Discussion title.
15. Title for Fig. 9 should be “Phage resistant mutants of *Mycobacterium smegmatis*”
16. Line 468: 5 ml drop seems do big to be used for this experiment. It must be a typographical mistake, correct it.
17. In the legends of Fig. 9 and Fig. 10 the phage is written as 19WeirdoES, match it to nomenclature used in the rest of the manuscript i.e. Weirdo19ES.
18. Include a color key of pham in Fig. 1.
19. Include the schematic of typical LysA with all the domain as a reference.
20. Images attached are blurred, provide good quality images for review.
